# Meta-transcriptomics characterization of individual *Marmota sibirica* reveals a wide spectrum of viral and bacterial pathogens in Inner Mongolia of China

**DOI:** 10.1128/spectrum.00180-25

**Published:** 2025-08-12

**Authors:** Min-Wu Peng, Yu-Hui Wu, Zi-Rui Ren, Narigen, Rui-Sen Hou, Gen-Yang Xin, Yu-Qi Liao, Jing Wang, Shi-Jia Le, Pei-Bo Shi, Hai-Long Zhao, Zi-Qing Deng, Da-Xi Wang, Chaolemen, Mang Shi

**Affiliations:** 1 National Key Laboratory of Intelligent Tracking and Forecasting for Infectious Diseases, School of Medicine, Shenzhen Campus of Sun Yat-sen University, Sun Yat-sen University 582261 , Shenzhen, China; 2 State Key Laboratory for Biocontrol, School of Medicine, Shenzhen Campus of Sun Yat-sen University, Sun Yat-sen University 582261 , Shenzhen, China; 3 Shenzhen Key Laboratory for Systems Medicine in Inflammatory Diseases, Shenzhen Campus of Sun Yat-sen University, Sun Yat-sen University 582261 , Shenzhen, China; 4 Old Barag Banner Center for Disease Control and Prevention, Hulunbuir, China; 5 BGI Research, Beijing, China; 6 Shenzhen Key Laboratory of Unknown Pathogen ldentification, BGl Research 213636 , Shenzhen, China; 7 College of Life Sciences, University of Chinese Academy of Sciences, Beijing, China; 8 Fujian Provincial Center for Disease Control and Prevention, Fuzhou, China; 9 Institute of Parasitic Disease Control and Prevention, Center for Disease Control and Prevention of Guangdong Province, Guangzhou, China; Changchun Veterinary Research Institute, Chinese Academy of Agricultural Sciences, Changchun, China

**Keywords:** metatranscriptomics, virome, pathogens, *Marmota sibirica*

## Abstract

Mongolian marmot is a reservoir for various zoonotic pathogens, including *Yersinia pestis*, *Bartonella*, and hantaviruses. However, our understanding of the marmot’s pathogen profile remains incomplete. Here, we used an unbiased metatranscriptomic approach to comprehensively characterize the virome and bacterial microbiome of 106 Mongolian marmot sampled from Chen Barag Banner, Inner Mongolian, China. A total of 12 mammalian-associated viruses from five families were identified, including novel species such as Chenqi marmot mastadenovirus 1 and Chenqi marmot enterovirus 1. Additionally, 62 species of picobirnaviruses were detected though they were not classified as mammalian viruses in this context. Co-infection was common, with an average of 1.35 mammalian viruses per marmot fecal sample, excluding picobirnaviruses. Phylogenetic analysis showed high homology between these viruses and those found in Himalayan marmots although some showing close relationship with rodent and pig viruses. Ribosomal binding site analysis suggested that 32 picobirnaviruses contained Shine-Dalgarno sequences, indicating potential bacterial association. Based on meta-transcriptomics sequencing data, our study also revealed five potential bacterial pathogens though *Yersinia pestis* was not found. In summary, this study provides a more comprehensive view of pathogen diversity in Mongolian marmots and contributes important insights to support disease monitoring and prevention efforts in border areas of China.

**IMPORTANCE:**

The Mongolian marmot (*Marmota sibirica*) is a critical mammalian species in Hulunbuir, playing a key role in harboring and transmitting *Yersinia pestis*, the causative agent of plague. However, research on the diversity, abundance, co-infection dynamics, and spillover potential of viruses and bacteria in these animals remains limited. This study characterizes pathogen diversity and ecology by examining the viral and bacterial microbiomes of Mongolian marmots in China’s northeastern border regions. The findings offer unique insights into potential threats to both animal and human health and contribute valuable data to inform disease prevention and control efforts in these regions.

## INTRODUCTION

The marmot, classified under the order Rodentia and belonging to the genus *Marmota*, encompasses a total of 14 species distributed across Eurasia and North America, inhabiting grasslands, plateaus, and mountainous regions (1). Primarily herbivorous, they live in colonies and possess strong digging capabilities, with some species exhibiting hibernation habits. In China, there are four main species of marmot: the Himalayan marmot (*Marmota himalayana*) found predominantly in Qinghai and parts of Tibet; the long-tailed marmot (*Marmota caudata*), the Mongolian marmot (*Marmota bobaksibirica*), and the gray marmot (*Marmota baibacina*), primarily distributed in Xinjiang, Tibet, Inner Mongolia, among other areas (2).

Marmot are known to host multiple pathogens that cause zoonotic diseases (3), including bacterial pathogens *Yersinia pestis*, *Coxiella burnetii*, *Ehrlichia* spp*.*, *Brucella* spp., and *Bartonella* spp. Additionally, the advent of metagenomic sequencing technology in recent years has led to an increasing number of reports on viral pathogens. For example, researchers have identified at least 18 genera belonging to 13 families of mammalian-associated viruses in Xinjiang’s long-tailed marmot (3). Similar discoveries were made for Himalayan marmot, which carry at least 15 genera across 9 families of mammalian-associated viruses, including astroviruses, bocaviruses, picornaviruses, and hepatitis E virus. Notably, picobirnaviruses are predominant in the intestinal tracts of Himalayan marmots, exhibiting high detection rates and close phylogenetic relationships with those identified in other mammals, while also displaying substantial sequence diversity (4). These findings contribute to a more comprehensive understanding of pathogen diversity across various marmot species.

The Mongolian marmot, also known as the Siberian marmot, is a key wildlife species on the Hulunbuir grasslands with significant public health implications. Epidemiological studies have linked human plague outbreaks to strains originating from Mongolian marmots, often associated with human contact during the skinning of marmots for meat and fur (5). Despite its public health significance, the infectome of Mongolian marmot remains poorly understood beyond *Yersinia pestis*. Previous studies on marmot viromes have primarily focused on Himalayan and Xinjiang long-tailed marmot, with limited attention given to the Mongolian marmot. However, expanding human activities—such as increased tourism to the Hulunbuir grasslands, domestication of Mongolian marmots as pets, and hunting for their meat and fur—significantly elevate the risk of zoonotic pathogen transmission between these marmots and humans (6). The application of metatranscriptomics, proven effective in uncovering novel pathogens across diverse sample types, holds great potential to deliver a comprehensive snapshot of the pathogen landscape in these mammals (7, 8).

Therefore, this study utilizes metatranscriptomic approaches to characterize major types of mammalian-associated virus and bacteria. The aim is to elucidate pathogen distribution in Mongolian marmots on the Hulunbuir grasslands, providing essential scientific insights for species conservation and public health risk assessment.

## RESULTS

### Sample distribution and characterization

**Fig 1:**
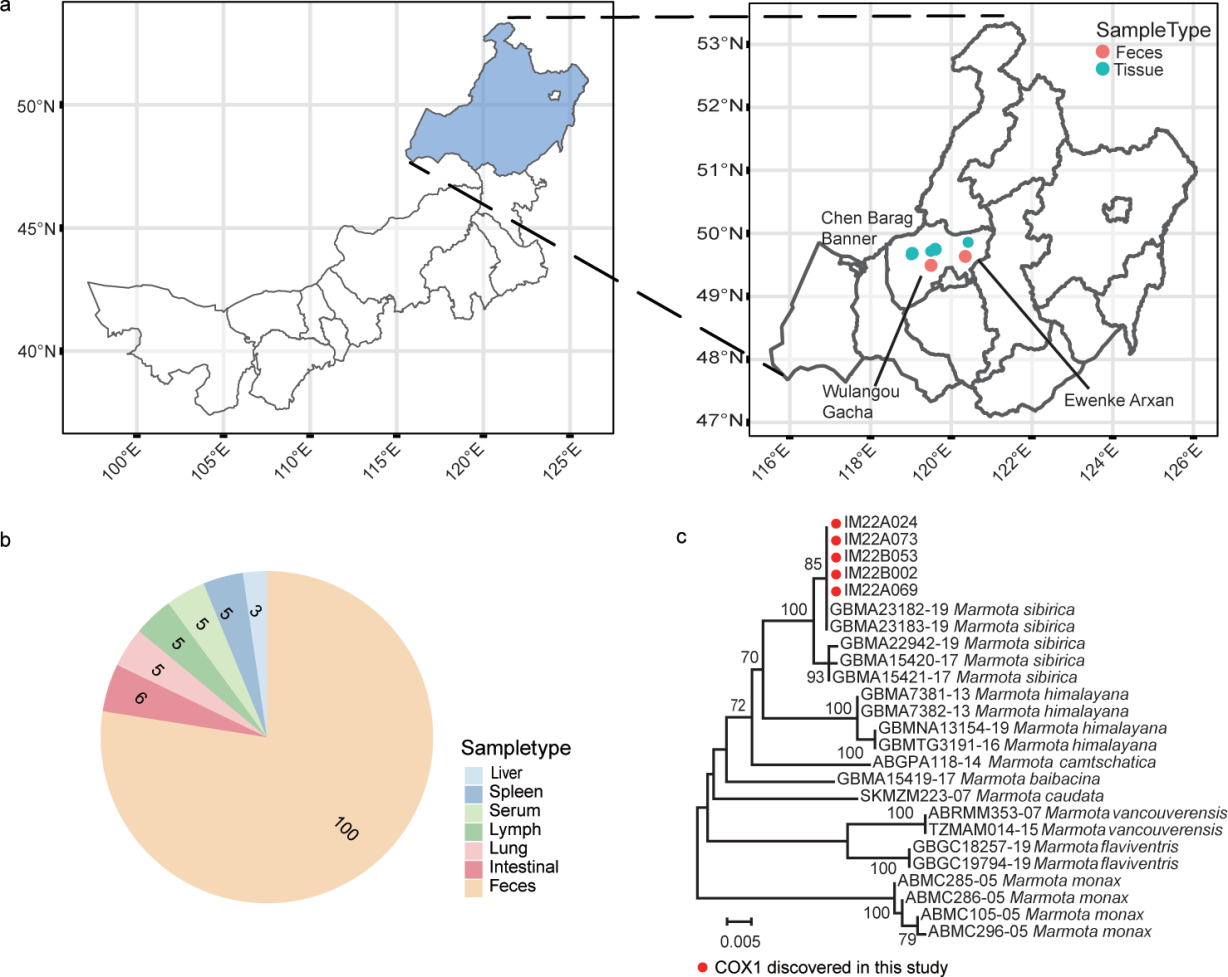
Overview of Sample Collection. (**a**) Sample distribution of marmots in this study. To ensure that each sample represents an individual marmot, only one fresh fecal sample is collected from each marmot family. The tissue samples were collected from marmots captured in two different locations. The basemap shapefile was generated in R using the SF package, with geographic data sourced from the publicly available GADM data set (https://gadm.org/download_country.html). (**b**) Number of libraries for seven sample types. (**c**) Marmot species identification based on the *cox1* gene. The reference sequences of *cox1* are download from Bold Systems v4 (https://v4.boldsystems.org/). The figure displays five representative *cox1* sequences, each derived from a distinct sample source and marked with a red dot.

This study was performed at Chen Barag Banner, Hulunbuir, Inner Mongolia Autonomous Region, which is located on the western edge of the Greater Khingan Range and bordering the Hulunbuir Plateau. Chen Barag Banner features mid-low altitude hills to the east and undulating highlands to the west, with elevations averaging 600–700 m. From May to June 2022, 100 fecal samples were obtained from Mongolian marmot at two locations: Ewenke Arxan Gacha and Wulangou Gacha (Fig. 1a). In May 2023, six Mongolian marmots were captured, two of which appeared to be in markedly poor physical condition. Liver, spleen, lung, lymph nodes, serum, and intestinal contents were collected from all individuals (Table S1; Fig. 1b). To verify the fecal samples origin, the cytochrome *c* oxidase subunit I (*cox1*) gene was used as a molecular marker. Phylogenetic analysis of the *cox1* gene (Fig. 1c) clearly differentiated among the four marmot species in China, validating that all samples in this study were derived from Mongolian marmots (9).

A total of 136 metatranscriptomic libraries were prepared, including 100 from fecal samples obtained from 100 individual marmots, and 36 from additional 6 marmots, each contributing liver, spleen, lung, lymph nodes, serum, and intestinal content samples. Successful sequencing was completed for all fecal samples and 29 tissue samples. Seven libraries from liver (*n* = 3), spleen (*n* = 1), serum (*n* = 1), lymph (*n* = 1), and lung (*n* = 1) were excluded from the analyses due to unsuccessful library construction or substantial contamination from unrelated sources. On average, each library generated 1.41Gbp of sequencing data (range: 0.018G-8.3G) and produced approximately 12,497,519 reads per library (range: 278,355–226,137,636). The sequencing reads were assembled into 95–1,873,327 contigs per library (Table S1), which were subsequently analyzed for pathogen discovery and characterization.

### Mammalian-associated virome characterization

Analysis of assembled contigs revealed a diverse range of DNA and RNA viruses. The study focused on characterizing mammalian-associated viral communities in Mongolian marmot, excluding viruses related to non-vertebrate animal hosts, dietary sources, parasite co-infections, or endosymbionts. In total, 12 mammalian-associated viral species were detected, including two DNA virus families, *Adenoviridae* and *Parvoviridae*, and two RNA virus families, *Astroviridae* and *Picornaviridae*. Among these are four novel and eight previously known virus species. The novel species were identified from the genus *Mastadenovirus* from the family *Adenoviridae* (*n* = 1), *Dependoparvovirus* of *Parvoviridae* (*n* = 1), *Enterovirus* (*n* = 1), and *Hunnivirus* (*n* = 1) from *Picornaviridae*.

**Fig 2:**
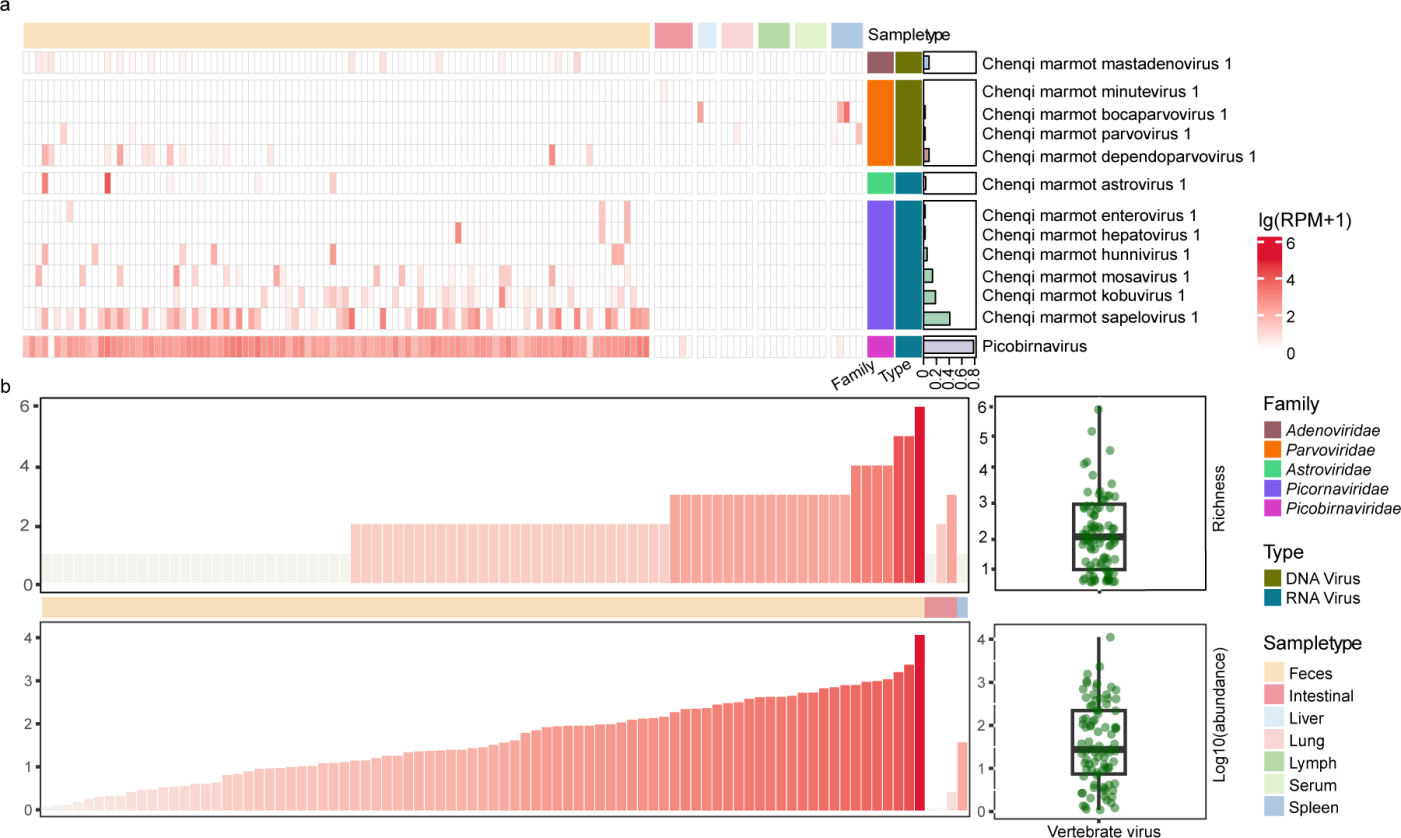
Mammal-associated viral diversity and abundance in Mongolian marmots. (**a**) Heatmap based on abundance of viruses and bacteria. The color of the annotations on the top panel represents different sample type, while the bar chart on the right panel represents the prevalence, and the color of the blocks in the heatmap represents the abundance of viruses. Viral abundance is visualized using a color scale based on log₁₀(RPM + 1), while viral prevalence is calculated as the number of positive individuals divided by the total number of individuals sampled. The heatmap is generated by ComplexHeatmap package (10) implemented in R. (**b**) Viral richness (top panel) and abundance (bottom panel) in each individual. Plot is generated by ggplot2 (ggplot2: Elegant Graphics for Data Analysis (3e)).

The prevalence of mammalian-associated viruses in the fecal samples varied. The *Picornaviridae* was present in 81% of individuals, including *Enterovirus* (4.72% of all individuals), *Hunnivirus* (8.49%), *Kobuvirus* (27.4%), *Mosavirus* (20.8%), *Hepatovirus* (4.72%), and *Sapelovirus* (52.8%). The positivity rate for the *Parvoviridae* is lower than *Picornaviridae* at 17.9%, with *Bocavirus* at 4.72%, AAV (Adeno-Associated Virus) at 16%, and *Parvovirus* at 6.6%. In the *Adenoviridae*, the positivity rate for Red squirrel dependoparvovirus is 17.9%. The prevalent rate for *Astroviridae* is the lowest at 6.6% (Fig. 2a; Table S2).

In addition to the confirmed mammalian viruses, a total of 62 *Picobirnaviridae* species were identified, including 59 novel species. Although their association with mammals remains unclear, picobirnaviruses (PBVs) were highly abundant, detected in 97% of individuals. Positivity rates for individual PBV strains ranged from 1% to 51%, with a median of 7%.

Compared to fecal samples, fewer viral species were identified in tissue samples (Figure X2). The most frequently detected viruses in the tissue were parvovirus, bocavirus, and PBVs. Bocavirus was found in spleen and liver samples, Parvovirus in spleen and lung samples, and a PBV in the spleen. No additional rodent tissue-associated viruses were detected.

### Ecological dynamics of the marmot viruses

This study quantified the abundance and diversity of viruses in each Mongolian marmot (Fig. 2b). On average, fecal samples from each individual contained a median of two mammalian-associated viruses (range: 1–6), and the average abundance was 259.25 RPM (median: 13.29, range: 0–11206.80). In tissue samples, each individual harbored a median of one mammalian-associated virus (range: 0–3). Co-infection is particularly common among *Picornaviridae*, such as *Sapelovirus* co-infected with *Kobuvirus* (8 cases) and mixed infection involving *Sapelovirus*, *Kobuvirus*, and *Mosavirus* (4 cases). The above co-infections were all detected from fecal samples.

### Evolutionary analysis of mammal-associated viruses

**Fig 3:**
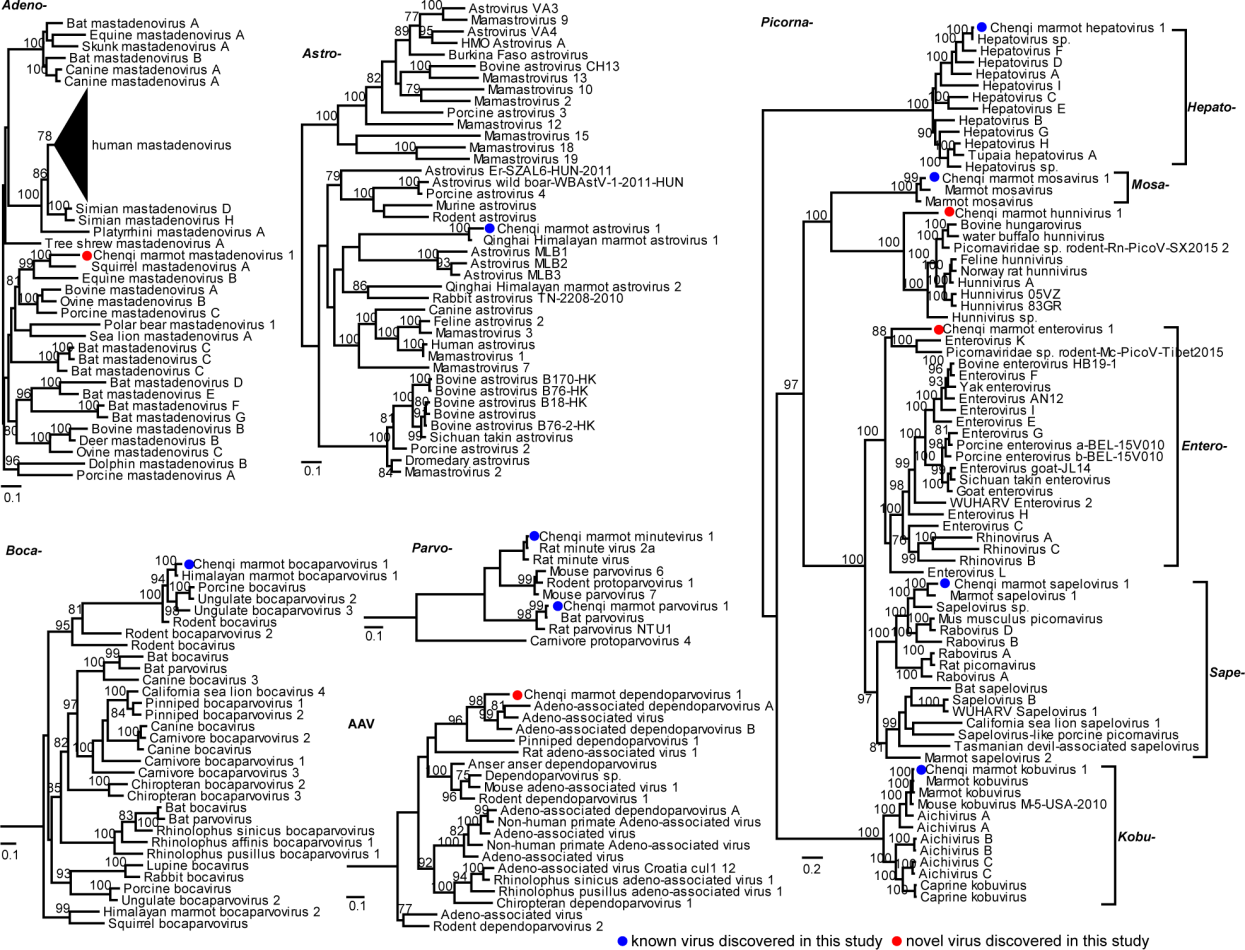
Phylogenetic relationships of viruses identified in this study with known viruses. Maximum likelihood phylogenetic trees are constructed at the family level based on conserved viral proteins: RNA-dependent RNA polymerase (RdRP) for RNA viruses, capsid protein 1 (VP1) for *Parvoviridae*, and hexon protein for *Adenoviridae*. All trees are midpoint-rooted for clarity. Red dots indicate previously described virus species in this study, while blue dots represent novel viral species. The trees (newick format) with complete information are provided in Figshare under the link https://figshare.com/articles/figure/Figure/29047028?file=54492887.

Phylogenetic analysis revealed that these newly discovered viruses from Mongolia marmots showed close relationship with those identified from other marmot species, especially with Himalayan marmots (Fig. 3). Specifically, 8 virus species were shared among these marmot species and 11 species between Mongolian and Himalayan marmots (Table S3). This included members of adenoviruses, parvoviruses, bocaviruses, astroviruses, and picornaviruses. For example, this study identified an astrovirus, designated as Chenqi marmot astrovirus 1 (Fig. 3), exclusively detected in fecal samples with a prevalence rate of 6.6%. The virus shared 88.39% nucleotide similarity with the Qinghai Himalayan Marmot Astrovirus 1 (HHMAstV1) strain previously isolated from intestinal samples of Himalayan marmots (11). Amino acid comparisons of its ORF1a, ORF1b (encoding RdRp), and ORF2 with HHMAstV1 revealed identities of 96.6%, 92.4%, and 93.0%, respectively. Additionally, the study identified a Bocaparvovirus species, named Chenqi marmot bocaparvovirus 1 (BOV), which was detected in one liver and two spleen samples. Based on VP1 similarity, it showed 97% nucleotide identity with Himalayan Marmot Bocaparvovirus 1 (HMBOV1) (12), previously isolated from the liver tissue of Himalayan marmots(13). And Kobuvirus species, named Chenqi marmot kobuvirus 1 (KOV), had 97.4% protein similarity with the related virus identified in the gut of Himalayan marmots. Other viruses are related to those infecting other mammalian hosts including rodents and domestic animals. For example, comparing the similarity of VP1 based on the Chenqi marmot minutevirus 1, it showed the highest amino acid identity with Rat minute virus 2a (RMV-2a) isolated from rats, reaching 98.5%. In addition, one *Protoparvovirus*, named Chenqi marmot parvovirus 1, was detected in one liver and two spleen samples. Based on VP1 similarity, it shows 97.9% nucleotide identity with bat parvovirus BtHp-PV/GD2012 strain, which is hosted by *Hipposideros Pomona* (14).

**Fig 4:**
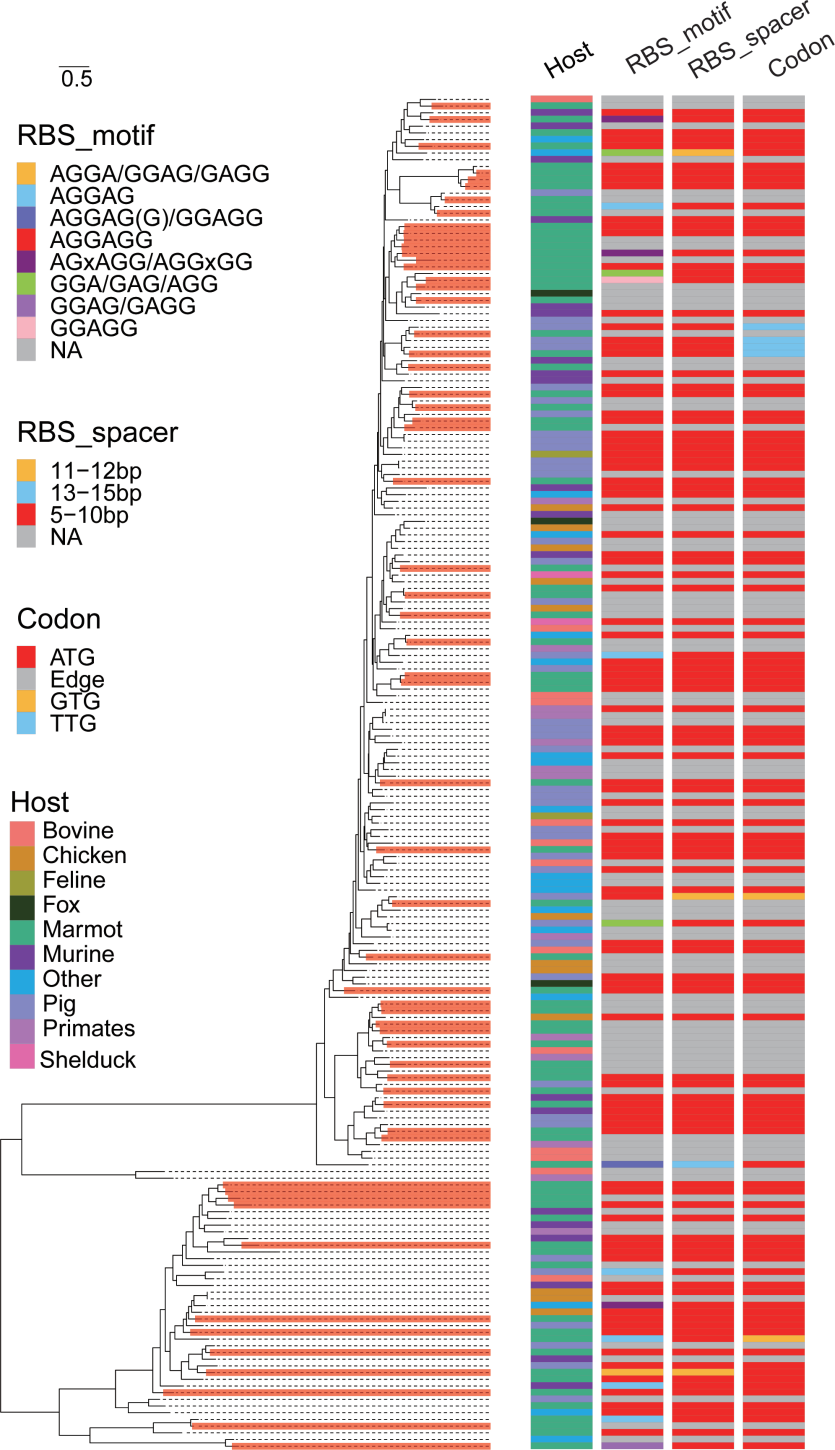
Characterization of picobirnaviruses discovered in this study. Maximum likelihood phylogenetic trees are constructed for *Picobirnaviridae*. The red bar represents the virus discovered in this study. Detailed information of each sequence, namely, host, RBS motif, RBS spacer, and codon are labeled on the right of the figure.

The study identified 61 PBVs (Fig. 4). The PBVs identified in this study were closely related to PBVs isolated from various species such as pigs, foxes, primates, and birds. BLAST analyses revealed amino acid identities ranging from 64.3% to 96.7% compared to PBVs in the NCBI database. Among these, 26 are closely related Himalayan marmot PBVs, with amino acid identities between 61.7% and 96.7%. Notably, only three PBVs exhibit amino acid identities exceeding 90% with previously identified Himalayan marmot PBVs.

Recent studies suggest that members of the *Picobirnaviridae* may be associated with bacteria (15, 16). In prokaryotic mRNA, the ribosome binding site (RBS) is located 8–13 nucleotides upstream of the start codon (e.g., AUG) and typically features a Shine-Dalgarno (SD) sequence (AGGAGG), which is complementary to the 3′ end of 16S rRNA in the 30S ribosomal subunit, promoting efficient ribosome binding (4, 17, 18). Predictive analysis revealed that 32 PBVs identified in this study contain the SD sequence (AGGAGG) approximately 5–10 bp upstream of the translation initiation site (AUG) of the RdRp gene. Notably, the actual number of SD-containing sequences is likely underestimated, as a substantial proportion of viral sequences lacked identifiable start codons and therefore could not be assessed for upstream SD motifs. This suggests that the true prevalence of SD-containing sequences among the viruses we identified may be higher than reported.

### Characterization of marmot gut microbiota

**Fig 5:**
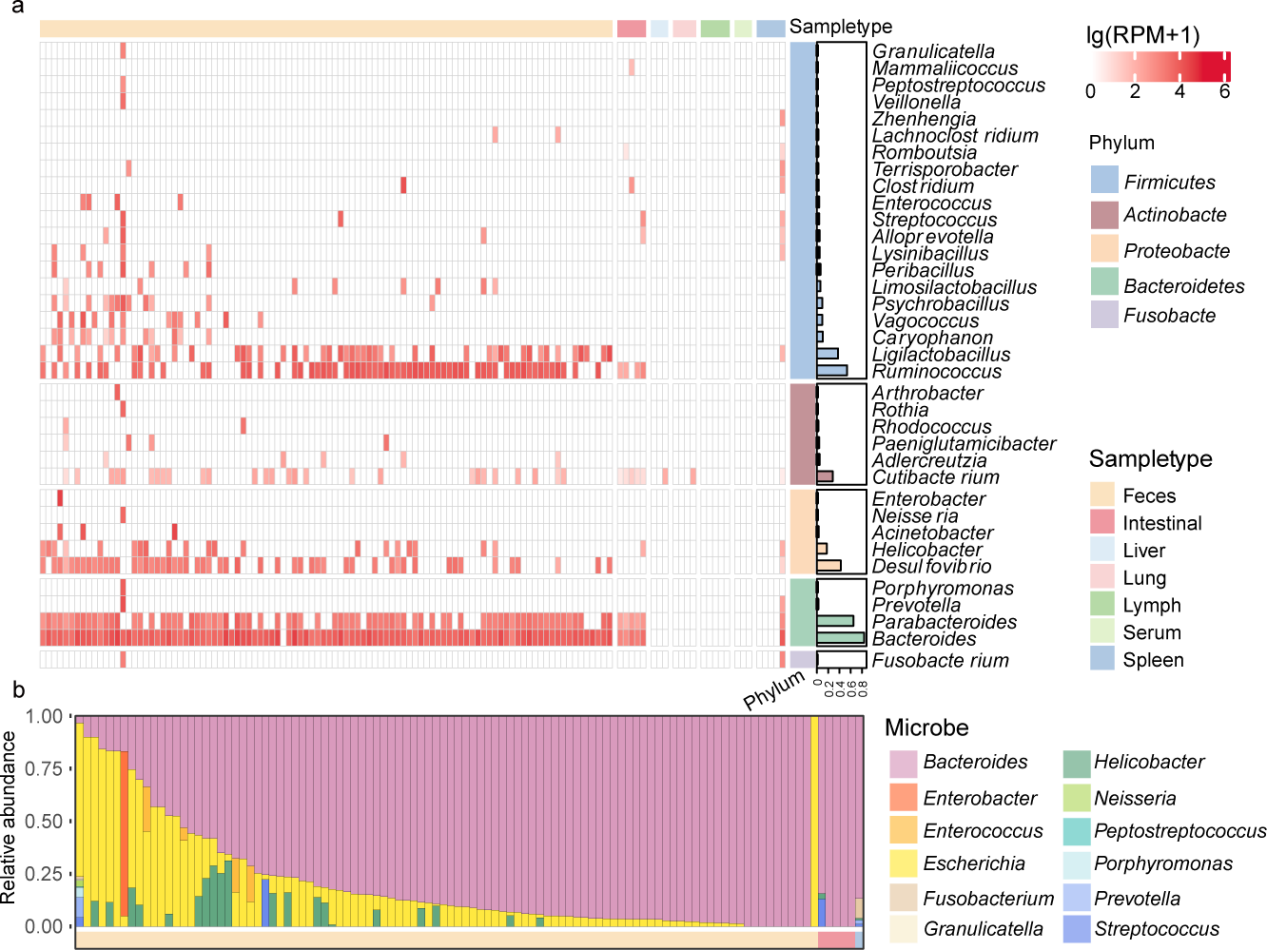
Taxonomic profile of gut microbiota at the genus level in Mongolia marmots. (**a**) The color annotations on the top panel represent different groups, while the bar chart on the right displays the prevalence. The color of the blocks in the heatmap indicates bacterial abundance. (**b**) Relative abundance of bacterial genera containing potential pathogens at the genus level.

We analyzed fresh fecal samples from Mongolian marmots to examine the composition of their intestinal microbial communities. The gut microbiota was found to be dominated by five phyla: Actinobacteria, Firmicutes, Bacteroidota, Proteobacteria, and Fusobacteria, which collectively constituting 80%–100% of the total microbial population. At the genus level, 37 genera were identified, with core members including *Bacteroides*, *Desulfovibrio*, *Escherichia*, *Helicobacter*, *Ligilactobacillus*, *Parabacteroides*, and *Ruminococcus* (Fig. 5a). These core genera play essential roles in digestion, such as cellulose degradation and polysaccharide decomposition. Among the bacterial genera, *Bacteroides* and *Escherichia* were predominant in individual marmots, with their combined relative abundance showing a median value of 75% (range: 20%–100%). Some genera, such as *Neisseria* (1/106), *Enterobacter* (1/106), *Streptococcus* (4/106), and *Acinetobacter* (4/106), were found only in a few individuals.

### Potential bacterial pathogens

**Fig 6:**
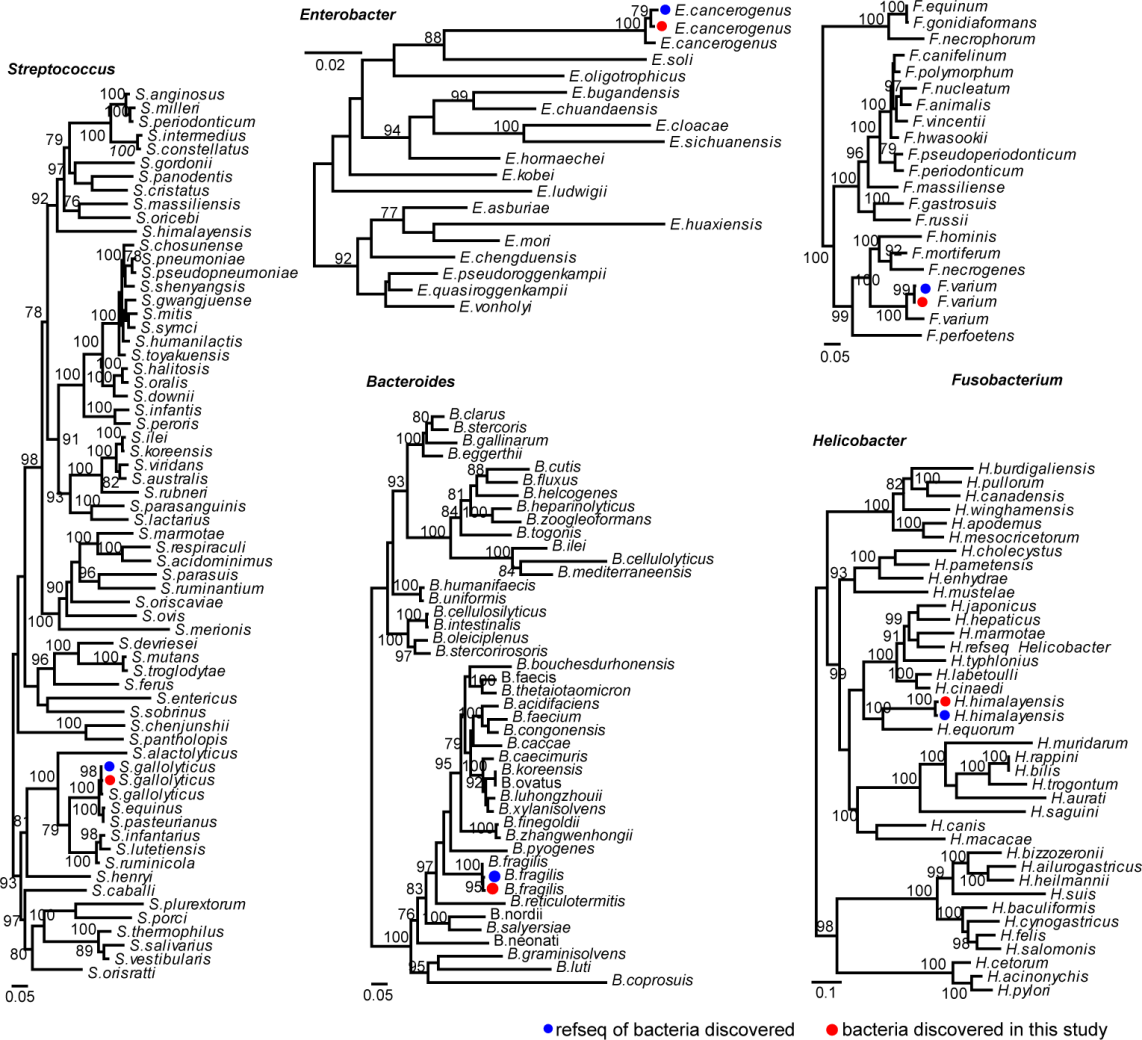
Marker gene-based identification of potentially pathogenic bacteria. Phylogenetic trees of potential bacterial pathogens were constructed using nucleotide sequences of the gyrA (alpha subunit of gyrase) or gyrB (beta subunit of gyrase) genes. The *Helicobacter* tree was built using gyrA, while the trees for *Bacteroides*, *Enterobacter*, *Fusobacterium*, and *Streptococcus* were based on gyrB sequences. All trees are mid-point rooted for clarity.

We also identify pathogenic bacteria species based on marker genes. Five potential pathogenic bacteria were identified, namely, *Helicobacter himalayensis*, *Bacteroides fragilis*, *Enterobacter cancerogenus*, *Fusobacterium varium*, and *Streptococcus gallolyticus* (Fig. 6), which are known to infect animals or humans. *Helicobacter himalayensis* (18–21), the most commonly detected pathogens here (21% positivity rate), was first reported two decades ago from Himalayan marmots. Its pathogenic mechanism remains unclear though it may contribute to gastrointestinal mucosal lesions. *Enterobacter cancerogenus*, identified in the spleen of a single marmot, is suspected to act as an opportunistic or secondary pathogen in conditions like pneumonia and wound infections. *Streptococcus gallolyticus*, associated with bacteremia, endocarditis, and colorectal cancer in human, had a 3.77% positivity rate. Finally, no *Yersinia* bacteria was identified in our data sets.

## DISCUSSION

The expansion of human activities, such as habitat destruction, hunting, and the domestication of wildlife, increases the risk of pathogen transmission from animals to humans (22, 23). To address this, it is crucial to investigate wildlife viruses, particularly in remote habitats like those of Mongolian marmots in China’s Hulunbuir grasslands. This study used metatranscriptomic sequencing to analyze tissue sample and fecal samples from 106 Mongolian marmots, identifying 12 virus species and 5 bacteria species that were likely to infect mammals. However, most of these pathogens are likely to associated with infections in animals but not humans, with the exception of *Helicobacter, Neisseria,* and *Streptococcus* bacteria, which might act as opportunistic pathogens in humans. The lack of detection of human pathogens such as *Yersinia pestis* may reflect the fact that the sampled Mongolian marmot populations were mostly healthy, thereby limiting the chance of pathogen presence. Alternatively, the sample type analyzed—primarily fecal samples—represents only certain pathogen types, while major pathogens like *Yersinia pestis* are more likely to be detected in serum or internal organs. Furthermore, the tissue samples we collected contained significantly fewer pathogens compared to fecal samples. In fact, only a limited number of pathogens were detected in the tissues, likely because tissues from healthy individuals are not expected to harbor high levels of viral pathogens. However, the current sample size (six individuals) is too small to draw definitive conclusions.

PBVs are among the most diverse viruses detected in our samples, but their host range remains debated. Commonly found in the feces and intestinal contents of humans and animals, PBVs are considered opportunistic enteric pathogens in vertebrates (17). This study identified highly divergent PBVs in fecal samples, clustering with PBVs from various hosts (birds, primates, rodents). However, 52.4% (32/61) of PBVs contained an SD sequence, supporting their phage-like nature, as previous studies have shown PBV SD sequences function in *Escherichia coli* (21, 24). In a recent study, Sadiq et al. not only identified Shine-Dalgarno (SD) motifs in PBVs but also compared host switching rates between *Picobirnaviridae* and other RNA viruses (25). They found that PBVs exhibit significantly higher rates of host switching, a feature more consistent with bacterial rather than eukaryotic hosts. Furthermore, SD motifs are markedly more enriched in PBVs than in other RNA bacteriophages. Collectively, these observations support the hypothesis that PBVs are more likely associated with prokaryotic hosts than with vertebrates. However, the reason for their high abundance in marmot species remains unclear.

Metatranscriptomics can be used identify not only viral pathogens, but also potential bacterial pathogens, and this has been applied to many other organisms such as humans, pigs, and sandflies (26). Here, we identified dominant viral and bacterial species within the fecal samples. Although bacteria dominated the samples, the data did not fully characterize the gut microbiota expression profile of individual marmots, with only a limited number (<5 genera) of bacterial communities detected in some individuals. This could be attributed to two factors: (i) insufficient sequencing data and (ii) the reliance on metatranscriptomics, where the majority of bacterial reads were associated with rRNA, revealing only the dominant bacteria such that other less abundant commensal bacteria are less well characterized here.

In Chen Barag Banner, four historical plague foci exist, three of which currently host large populations of Mongolian marmots. Recent years have seen sporadic human cases of plague, including two in Mongolia in 2019 and three in 2020, all involving local herdsmen (27, 28). Animal plague outbreaks have been increasing in Inner Mongolia, with severe transmission intensity and widespread impact (28). Additionally, Daurian ground squirrels are encroaching on Mongolian marmot habitats, potentially replacing them as primary plague hosts. Although *Yersinia pestis* was not detected in the collected organ samples, including mostly feces and a few serum samples, the region’s proximity to natural plague zones in Russia’s Transbaikal and eastern Mongolia, combined with increasing tourism and international exchanges under the “Belt and Road” initiative, underscores the importance of enhanced plague monitoring and preventive measures in both animals and humans.

## MATERIALS AND METHODS

### Specimen collection and processing

Fecal samples from Mongolian marmots were collected between May and June 2022 at two locations in Chen Barag Banner, Hulunbuir City, Inner Mongolia, China. A total of 100 fresh fecal samples were obtained, with only one sample collected per marmot burrow to ensure individual representation. Samples were immediately preserved in 1 mL of RNA later reagent upon collection. In May 2023, six Mongolian marmot were captured in Chen Barag Banner using trapping methods. The samples, including liver, spleen, lungs, lymph nodes, intestinal contents, and serum, were promptly dissected in the local CDC laboratory and transported on dry ice to a −80°C freezer. All protocols for sample collection and processing were reviewed and approved by the Ethics Committee of Sun Yat-sen University (SYSU-IACUC-MED-2021-B0123)

The fecal and tissue samples were mixed with 600 µL of RLT buffer containing beta-mercaptoethanol in a grinding tube and then homogenized using an electric tissue grinder in an ice bath. Total RNA was extracted and purified with the RNeasy Plus Universal Mini Kit (QIAGEN, Germany). RNA quality was assessed using a NanoDrop One microvolume spectrophotometer. The extracted RNA was transported on dry ice to BGI Genomics, where library construction and sequencing were performed. For each sample, sequencing libraries were constructed using the MGIEasy RNA Library Prep Kit V3.0 (BGI, China). The libraries were subsequently sequenced on the DNBSEQ T series platform (MGI, China), generating 150 bp paired-end metatranscriptomic reads, with a target yield of 50 Gbp.

### Virus discovery and confirmation

For each library, once the sequencing data were obtained, quality control of the reads was initially conducted using bbduk.sh (https://sourceforge.net/projects/bbmap/; parameters, maq = 10 qtrim = r trimq = 10 ftl = 1 minlen = 90) (Version 39.06). rRNA reads were removed by aligning the short sequences against the rRNA database downloaded from the SILVA website (https://www.arb-silva.de/) using Bowtie2 (Version 2.5.3), using the “--very-fast-local” option (29). The remaining reads were *de novo* assembled using MEGAHIT (Version 1.2.8) (30) with default parameters. The assembled contigs were compared to the NCBI non-redundant protein database (nr) using Diamond blastx (Version 2.0.14.152) (31). An *E*-value cutoff of 10^−4^ was set to maintain high sensitivity while reducing false positives. The taxonomic lineage information for the best hit of each contig was obtained, and those classified under the viral domain were considered potential viral genomes. The final viral genomes were validated by mapping the reads back to the corresponding contigs and examining the mapping results using Geneious (Version 9.1.4) (32). Erroneous regions in viral contigs were identified and trimmed through manual curation informed by read mapping results, followed by efforts to merge contigs corresponding to the same viral species.

Among the viral contigs described here, those potentially associated with mammals, particularly vertebrate-specific viruses and vector-borne vertebrate viruses, were preliminarily identified based on the taxonomic lineage information from the Blastx results and confirmed through phylogenetic analysis. Viral-like contigs were processed by trimming host-derived sequences from both ends using VirSorter2 (v2.2.4) with default databases and parameters (33). Viral contigs that belonged to vertebrate-specific or vector-borne viral families/genera/species were subsequently identified as vertebrate viruses. The virus species demarcation was conducted following the species demarcation criteria established by the International Committee on Taxonomy of Viruses (ICTV, https://ictv.global/) (Table S4). For genera without established species demarcation criteria, we applied a stringent threshold of 90% amino acid identity in the RdRP to known viral species. This threshold was also used to define novel species.

### Evolutionary analysis

To elucidate the evolutionary history of all vertebrate-associated viruses, this study initially assigns these viral sequences to their respective virus families or genera (associated with vertebrates) based on Diamond Blastx results. For interspecies phylogenetic analysis grounded in conserved protein alignments, it utilizes the amino acid sequences of the RNA-dependent RNA polymerase (RdRp) for RNA viruses and the replicase or DNA polymerase (DNA pol) for DNA viruses. Subsequently, employing the L-INS-I algorithm in MAFFT (Version 7.480) (34), the conserved proteins of these viral genomes are aligned with related viral genomes downloaded from GenBank. Post-sequence alignment, TrimAL (Version 1.2) (35) is used to excise all ambiguous alignment regions. The maximum likelihood method provided in PhyML (Version 20120412) (36), featuring the LG model of amino acid substitution and the subtree pruning and regrafting (SPR) branch-swapping algorithm, is then utilized. Support for specific nodes on the trees was evluated using an approximate likelihood ratio test (aLRT) with the Shimodaira-Hasegawa-like procedure.

To confirm that the fecal samples are from Mongolian marmots, molecular identification of the host is carried out. Initially, contigs annotated as marmot mitochondrial cytochrome *c* oxidase subunit I (*cox1*) sequences were screened using Blastx. These contigs were then compared to the BOLD database (http://www.boldsystems.org/), and those with a 98% match to reference sequences were considered conspecific. A phylogenetic tree is constructed to determine the position of the sequences within the tree.

### Quantification of viral abundance

In this study, the abundance of each virus in every library was quantified as the number of viral reads per million non-rRNA reads by mapping the non-rRNA reads of each library to their corresponding viral genomes. To mitigate false positives, the study employed the bbmask tool (http://sourceforge.net/projects/bbmap/) to mask low-complexity regions and applied an abundance threshold of RPM (Reads Per Million) > 1, where RPM is calculated as Virus reads count/Total non-rRNA reads × 10^6^. This threshold, validated in prior publications, ensures a low false positive rate. To eliminate index hopping artifacts introduced during sequencing, a threshold of 0.1% of the highest read count for each virus species within the same sequencing lane was set; reads below this threshold were deemed “negative.”

### Identification and quantification of bacteria

For bacterial analysis, this study quantified the abundance of each genus in every library by mapping the non-rRNA reads to the rRNA-depleted GTDB database (https://gtdb.ecogenomic.org), expressing the bacterial load as reads per million non-rRNA reads (RPM). To minimize false positives, the metaphlan4 program (version 4.1.0) (37) was employed for confirming bacterial genera and to estimate the abundance levels for each bacteria genus. Genera with genome coverage not less than 0.1% and present in both outputs were deemed reliable. For higher quality bacterial contigs, a mixed-library assembly was performed based on sample type, aiming to obtain the complete conserved genes. In this context, potential pathogens were classified to the species level using phylogenetic analysis based on the rpoB (RNA polymerase subunit beta) (38) and gyrB (gyrase subunit beta) (39) genes, while non-pathogenic microorganisms were classified to the genus level.

### RBS analysis

For ribosome-binding site (RBS) prediction, the tool Prodigal (version 2.6.3) (40) was employed. Custom scripts were used to extract the “rbs_motif” field from the GFF output file and classify different 5′ UTR sequences into “SD” (for conserved motifs resembling AGGAGG, the classical Shine-Dalgarno), “none,” and “other.”

## Data Availability

All sequencing data have been deposited in the NCBI Sequence Read Archive (SRA) under BioProject ID PRJNA1238520, as well as in the China National Genebank (CNGBdb) under accession CNP0004142. The viral genome sequences generated in this study have been deposited in the National Genomics Data Center (NGDC) under accession numbers C_AA108212.1-C_AA108285.1.

## SUPPLEMENTAL MATERIAL

Table S1 spectrum.00180-25-s0001.xlsx Library information.

Table S2 spectrum.00180-25-s0002.xlsx Pathogens summary.

Table S3 spectrum.00180-25-s0003.xlsx Comparisons of virome between different marmot species.

Table S4 spectrum.00180-25-s0004.xlsx Virus blast results and species demarcation criteria in ICTV.

ASM does not own the copyrights to Supplemental Material that may be linked to, or accessed through, an article. The authors have granted ASM a non-exclusive, world-wide license to publish the Supplemental Material files. Please contact the corresponding author directly for reuse.
